# 186. Characteristics of COVID-19 Vaccine Breakthrough Cases in Minnesota, 2021

**DOI:** 10.1093/ofid/ofab466.186

**Published:** 2021-12-04

**Authors:** Stephanie Meyer, Amy Saupe, Aaron Bieringer, Corinne Holtzman, Luke Magnuson, Erica Bye, Miriam Muscoplat, Xiong Wang, Alexandra Lorentz, Kathryn Como-Sabetti, Ruth Lynfield, Ruth Lynfield

**Affiliations:** Minnesota Department of Health, Minneapolis, Minnesota

## Abstract

**Background:**

Over 600,000 COVID-19 cases, including >7000 deaths reported to MN Dept of Health (MDH) by June 1, 2021. Clinical trials demonstrated high effectiveness of COVID vaccines. We assessed COVID-19 cases among fully vaccinated residents [vaccine breakthrough (VB) cases].

**Methods:**

COVID-19 VB cases were MN residents with completed COVID-19 vaccination series ≥14 days prior to symptom onset or positive for SARS-CoV-2 by nucleic acid amplification or antigen test. COVID-19 cases were reported to MDH and COVID-19 vaccinations reported to the MN Immunization Information Connection (MIIC). COVID-19 cases were matched to MIIC to identify VB and interviewed; medical records of hospitalized cases were reviewed. Available VB case specimens underwent whole genome sequencing (WGS) at MDH or collaborating lab.

**Results:**

Jan 19 – June 1, 2021, 2765 VB cases were reported among >2.45 million fully vaccinated residents and 147,445 COVID-19 cases. VB case median (MED) age was 52 y (IQR 38, 68), 83% white, 65% female; MED age of fully vaccinated was 55 y (IQR 30, 68), 77% white, 54% female. Of VB cases, 273 (10%) were hospitalized and 32 (1%) died (MED age 74 y; IQR 66, 85). 2212 (80%) VB cases were interviewed; 60% reported symptoms; most common were fatigue (53%), rhinorrhea (49%), cough (42%), headache (41%). 35% reported a comorbidity.

Of hospitalized VB cases, 120 had completed record reviews. 64 were admitted for COVID-19 related illness (MED age 74 y, IQR:65, 83) including 27 admitted to ICU (MED age 71 y, IQR: 65, 83). 90% (108) reported a comorbidity, most common being chronic metabolic conditions (46%), obesity (45%), renal disease (31%) and chronic lung disease (26%); 27 were immunocompromised (not mutually exclusive), including immunosuppressive therapy (15), hematological malignancy (9), other cancer (11), and organ transplant recipients (8).

Of 604 VB case specimens, 79% were B.1.1.7, 9% B.1.427/429, 3% P.1, and 2% B.1.351; lineage distribution was similar to overall 24,157 MN SARS-CoV2 WGS data.

**Conclusion:**

Identified VB cases were 0.1% of those vaccinated and < 2% of total cases reported in the time period. COVID-19 vaccines are an important tool in preventing COVID-19. Additional surveillance, including WGS and case characteristics will be useful to monitor VB.

**Disclosures:**

**Ruth Lynfield, MD**, Nothing to disclose

